# Fluid management in extensive liposuction: A retrospective review of 83 consecutive patients: Erratum

**DOI:** 10.1097/MD.0000000000013212

**Published:** 2018-11-02

In the article, “Fluid management in extensive liposuction: A retrospective review of 83 consecutive patients”,^[1]^ which appears in Volume 97, Issue 41 of *Medicine*, the corresponding author information should appear as:

Tian-Lan Zhao, Department of Plastic Surgery, The Second Affiliated Hospital of Soochow University, 1055 Sanxiang Street, Suzhou, Jiangsu 215004, China (e-mail hezhenzhou1@126.com).

Wei-Gang Cao, Department of Plastic and Reconstructive Surgery, Shanghai 9th People's Hospital, Shanghai Jiao Tong University School of Medicine, 639 Zhi Zao Ju Road, Shanghai 200011, P.R. China (e-mail wgcao@sina.com).
